# Understanding the Adaptive Evolutionary Histories of South American Ancient and Present-Day Populations via Genomics

**DOI:** 10.3390/genes12030360

**Published:** 2021-03-02

**Authors:** John Lindo, Michael DeGiorgio

**Affiliations:** 1 Department of Anthropology, Emory University, Atlanta, GA 30322, USA; 2 Department of Computer and Electrical Engineering and Computer Science, Florida Atlantic University, Boca Raton, FL 33431, USA

**Keywords:** ancient DNA, natural selection, South America

## Abstract

The South American continent is remarkably diverse in its ecological zones, spanning the Amazon rainforest, the high-altitude Andes, and Tierra del Fuego. Yet the original human populations of the continent successfully inhabited all these zones, well before the buffering effects of modern technology. Therefore, it is likely that the various cultures were successful, in part, due to positive natural selection that allowed them to successfully establish populations for thousands of years. Detecting positive selection in these populations is still in its infancy, as the ongoing effects of European contact have decimated many of these populations and introduced gene flow from outside of the continent. In this review, we explore hypotheses of possible human biological adaptation, methods to identify positive selection, the utilization of ancient DNA, and the integration of modern genomes through the identification of genomic tracts that reflect the ancestry of the first populations of the Americas.

## 1. Introduction

**Figure 1:**
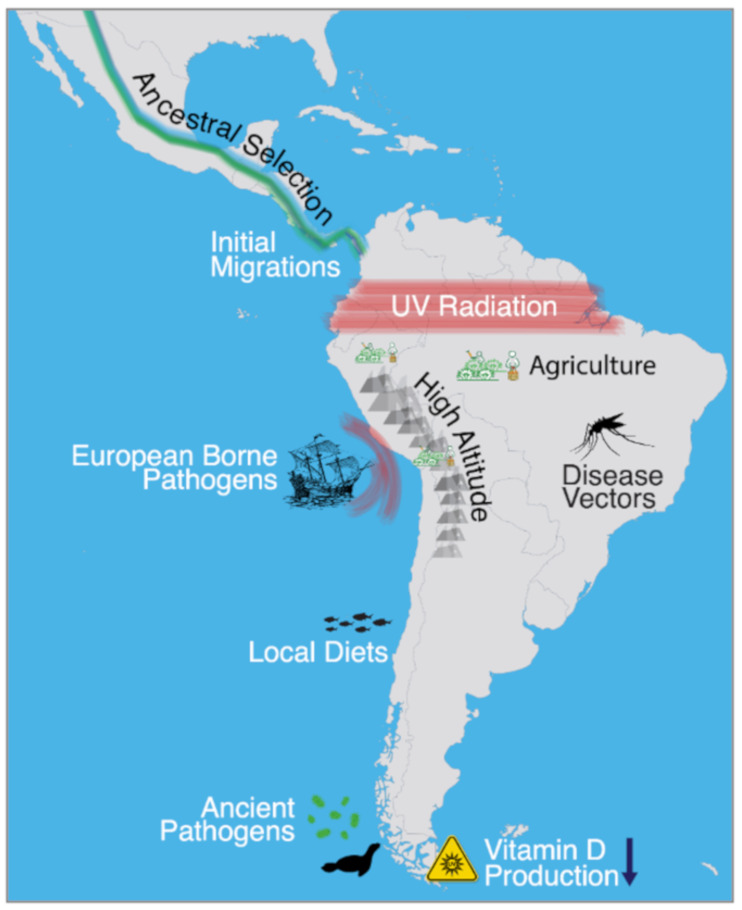
Environmental factors that may have posed adaptive pressures to the indigenous peoples of South America, including the arrival of Europeans in the 16th century.

When the first people arrived in South America, many thousands of years ago, they encountered a wide range of environments that greatly differed from their migratory point of origin: Beringia. The oldest archeological site on the continent, Monte Verde in present-day Chile, dates back to approximately 14,000 years ago [1], suggesting that humans had reached the continent rapidly after the initial entry into the Americas and splitting between North and South American lineages, some 17 to 14 thousand years ago [2,3]. By 10,000 years ago, archeological evidence suggests that humans were widespread throughout the continent, and were inhabiting some of the harshest environments, including the high-altitude Andes [4], the tropical regions of Brazil [5], and the subpolar region of Tierra del Fuego [6]. The successful establishment of populations within these diverse ecologies may have prompted natural selection to drive the rise of adaptive phenotypes, which may have involved diet, ultraviolet (UV) radiation, low-oxygen environments, and pathogens. Depending on the ecology, some environmental factors are better candidates than others for correlating with positive selection, especially regarding hypotheses involving pathogens (Figure 1).

While the archaeological record can provide clues about the cultures of the first people of South America, the use of ancient genomics has proven powerful in its greater resolution for understanding ancient migrations and establishing genetic continuity between ancient and living populations. Moreover, ancient genomics has the power and potential to refine our understanding of how the first settlers successfully established populations via adaptive evolutionary events. This power can be utilized to discover ancient pathogens, which are strong motivators for natural selection, and may have prompted local biological adaptation. A plethora of other known environmental factors may have also promoted adaptive responses (Figure 1), including cultural manifestations evidenced in the archaeological record, such as the rise of agriculture.

Perhaps one of the most confounding aspects of understanding adaptation in the Americas stems from the lack of knowledge surrounding the pathogens that the first settlers likely encountered. Contrary to notions of pristine environments before the arrival of Europeans, the early populations likely dealt with pathogens that may have prevented initial settlement success without adaptation. This scenario is likely in regions where disease vectors are present, such as mosquitos, or in tropical regions where the risk of parasitic infection is high. On the other hand, ancient skeletons have repeatedly shown disease indication that can be gleaned from disfigured morphology. These skeletons can harbor the microbial genomes that caused such pathologies and thus provide a window into the ancient pathogen landscape that existed before European contact.

Remarkably, the “omics” revolution can also yield insights into the changes that may have accompanied major social and cultural transitions that are coded in the epigenome, which meditates communication between the environment and the genome. These changes could be associated with simple differences between mobile and sedentary populations, or major cultural shifts that may have occurred after the advent of stable food sources from agriculture.

In this review, we will focus on various computational methods that can help leverage information from ancient populations, discuss hypotheses where natural selection may have been a factor, and discuss the possibility of exploring the changes that may have occurred in the lifetime of individuals in response to specific cultural conditions. Lastly, we will also discuss more recent adaptation in living populations that may have been the result of admixture with migrant populations after European contact.

## 2. High-Altitude Adaptation

From a genetic perspective, hypoxia adaptation is perhaps the most well-studied human trait in South America [7,8,9]. However, these studies have mainly focused on modern Peruvian populations, which have been affected by the bottlenecks that ensued upon European contact [8,9,10]. Furthermore, despite strong signals of positive selection detected in other high-altitude populations that relate to the hypoxic pathway [11,12], the highlanders of Peru do not exhibit the same signals, suggesting different adaptive pathways, which may include cardiovascular components [8]. Theoretically speaking, positive selection signals may be dampened in Andean highland populations due to population collapses that occurred after European contact in the region, which may have been as high as 80% according to historical estimates [13]. Collapses of such magnitude may have distorted allele frequencies as populations recovered, obscuring genomic signals related to high-altitude adaptation.

**Figure 2:**
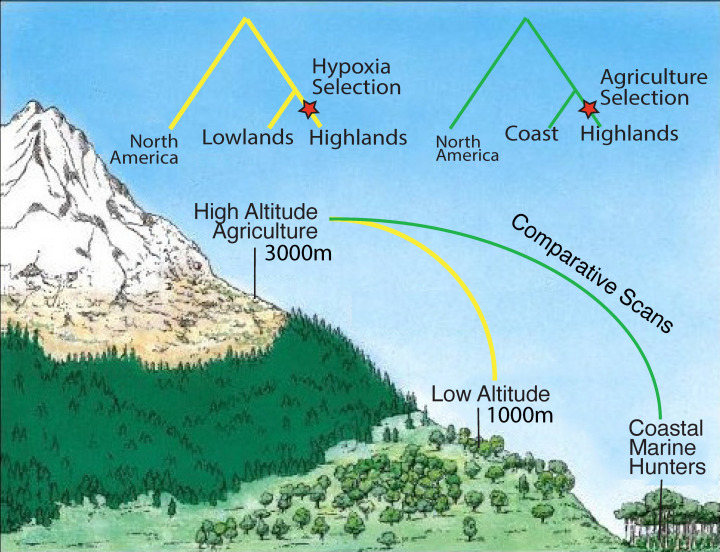
Detecting positive selection in the Andean highlands.

Future research may utilize ancient DNA before the population collapses to circumvent this potential issue. A gold standard study could employ DNA from individuals sampled in pre-contact populations from closely related Andean populations both from high-altitude and low-altitude regions (Figure 2). These populations were likely trading with each other given their proximity [14], and admixture between them would also need to be assessed and accounted for to avoid false-negative results. A new approach for detecting positive selection that may be particularly powerful for dealing with this type of data involves the joint assessment of admixture and allelic differentiation. The method, known as Ohana [15], could detect variants that deviate strongly in the high-altitude population from a genome-wide covariance structure created using both coastal and high-altitude populations. We describe this method further in Section 6.

## 3. Ultraviolet Radiation

Another factor that may have posed an adaptive challenge for the first settlers of the continent may be linked to ultraviolet (UV) radiation. While constant exposure in equatorial regions may have certainly proved difficult in this regard, settlement regions that are both equatorial and high-altitude may have been especially harsh, given the reduced atmospheric density absorbing less of the intense radiation. These regions would encompass the highlands of Colombia, Ecuador, and Peru, which have some of the highest UV radiation exposures in the world [16]. For those populations that inhabited the Andean highlands for thousands of years, variants in melanin genes may have become positively selected and differentiated to protect against folate damage [17]. Folate has been implicated in a variety of crucial cell pathways, including cell division, DNA repair, and embryonic development [17]. Extreme UV radiation, as would be expected in equatorial high-altitude regions, is thought to risk the photolysis of folate via the skin, and natural selection may have favored skin pigmentation that could help block harmful radiation in these types of environments [17].

Comparative genomic scans may hold the power to reveal selection on genes or pathways related to adaptation to excessively high UV radiation. For example, in sharp contrast to the traditional environment experienced by the Andean highlanders are those of the subpolar regions of Tierra del Fuego. This type of environment may have distinct selection pressures driven by conversely deficient UV radiation. Positive selection on depigmentation to increase UV exposure for vitamin D production may have been an adaptive factor for these subpolar populations. Vitamin D is important for both immune system support and fertility [18], which make significant deficiencies of this secosteroid a target for natural selection. However, these types of studies should also take into account the possibility of ancient selection that may have occurred before the migrations into the Americas or false-positive signals that may result from admixture after European contact [19].

## 4. Adaptive and Plastic Responses to Culture

Due to the genetic continuity that has thus far been revealed about the ancient cultures of South America (unlike Europe—where population replacements were an ongoing process [20]), the continent represents a rare opportunity to explore how populations adapted to cultural shifts from genomic and epigenomic standpoints. Specifically, there are two major shifts that can be explored, given the availability of viable samples. First is the transition from mobile to sedentary societies. This transition is observed in the archeological record from populations all over the world, yet little is known about the impacts of such a major shift for our species. For instance, questions arise as to whether neurological changes were needed to adapt to such a shift. Did genes that are associated with sociality in mammals, such as oxytocin and vasopressin [21], come under the specter of selection to facilitate the development of these societies? Did factors like close proximity between non-kin in urban centers drive this type of selection? On the other hand, did plasticity play a factor? Would we notice epigenetic changes along genes associated with behavior as humans made such major transitions in order to increase tolerance of each other? Recent advancements in ancient epigenomics has made pursuing such questions possible by computationally taking advantage of the features of post-mortem DNA damage to assess the probability of methylation at a cytosine site [22]. Ancient populations from the Andes present the perfect opportunity to conduct such a study, given repeated findings of genetic continuity through various cultural transformations [7,23,24]. However, other regions of South America remain unexplored via such time transects, including regions of Colombia, where archeological sites have yielded ancient individuals sampled through time [25].

The second major cultural transition that is nearly ubiquitous across global populations is the one to agriculture. Once again, the Andes present an ideal focal region as it is one of the main hubs of agricultural development on the planet. In general, diet is likely a trait that has come under strong positive selection in various populations through time, since it is tightly linked with survival and reproduction. However, agriculture marks a major deviation from evolutionary strategies in the past that likely relied on a variety of foods, probably compounded by location and seasonality. Agriculture marks the major shift in this trend, as food sources and diversity may have become limited. Famously, this has been observed in other parts of the world, evidenced by signatures of positive selection on variants associated with lactase persistence [26]. A glimpse of this adaptation has already been detected in the Lake Titicaca region of the Andes, where a variety of tubers were first domesticated some 6000 years ago, including the potato [27]. In this particular case, populations may have adapted to a consistent increase in starchy foods via a gene responsible for the final stages of starch digestion in the duodenum [7]. Also of note are previous notions that human copy number variation on amylase genes, associated with salivary starch digestion, are thought to be under selection in European populations [28]. This variation was not detected in the Andean populations despite the starch-heavy agriculture. However, recent population-level whole genome studies have demonstrated that copy number variation in European populations is highly variable and may not be a consequence of selection due to the advent of agriculture [29], while functional molecular studies have revealed no benefit for additional copy number with respect to starch digestion [30].

## 5. Ancient Pathogens and European-Borne Disease

**Figure 3:**
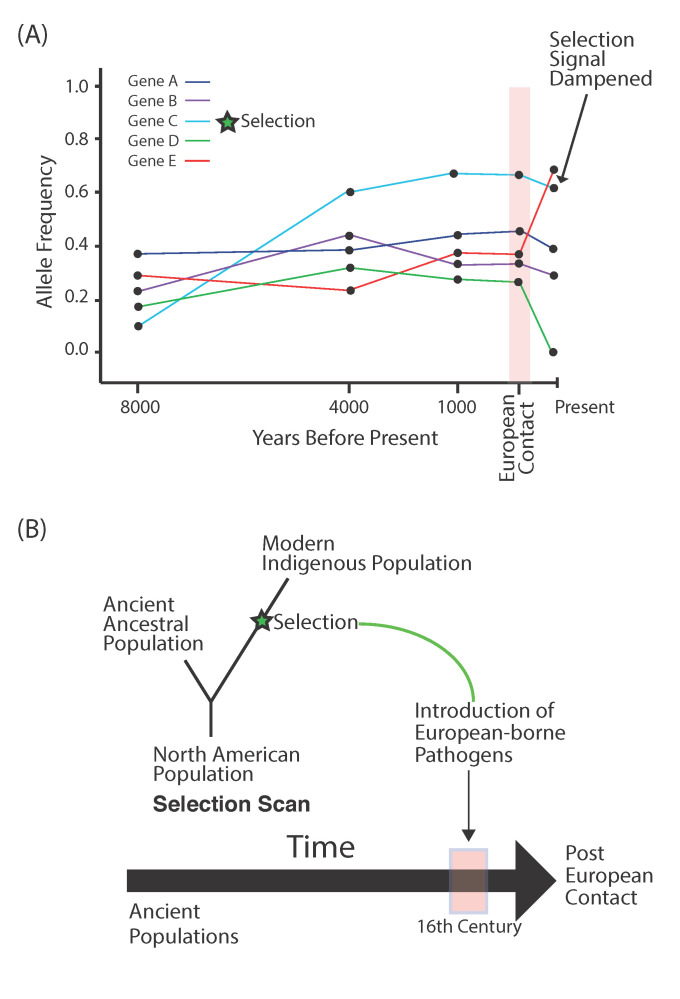
European contact and detecting selection. (**A**) The population collapse after European contact may obscure ancient signals of selection by causing variants not under selection to rise to high frequency stochastically. (**B**) Design to detect positive selection in response to European-borne pathogens.

Much speculation and theory has been associated with the pathogen loads that existed in the Americas. Upon entry into North America, new pathogens not present in Eurasia may have been encountered early, possibly prior to the population splits across South America [31]. However, it is likely that local ecologies and later specific sustenance strategies exposed populations to differential pathogen risk, such as those from agricultural vs. nomadic lifestyles. Ancient populations from South America present a rare opportunity to test adaptive evolutionary hypotheses in humans by juxtaposing closely related populations (potentially stemming from a single migration into South America) who underwent distinct cultural adaptations across environments. However, populations from South America suffered their greatest onslaught from European contact, where several pathogen strains were introduced in a short period of time [32]. While the extent of the collapses across various populations after European contact is debated with estimates varying wildly [13], there is little doubt that European-borne pathogens played a part. To further complicate the story, disease resistance was less likely in indigenous populations that suffered multiple epidemics at once, which may have overwhelmed their immune systems [32]. And yet, some indigenous populations survived, and this resilience may have been, in part, due to positive selection on variants that may have conferred some resistance to certain pathogens. One such scenario has been found in the high-altitude population of the Aymara in modern-day Bolivia. Not only was the ancient population collapse in this region found to be less severe compared to other parts of South America, but the population also shows patterns consistent with positive selection on variants that may be involved in the immune response to smallpox [7]. Evidence for positive selection was identified by utilizing ancient populations that showed genetic continuity with the living populations. In doing so, selection was detected along the branch of the living population, which diverged from their ancestors prior to European contact (Figure 3B). This type of technique may be used in the future to reveal further disease resilience in other indigenous populations.

There is also the prospect of adaptive gene flow. The theory here aims to examine indigenous populations that began to admix with differentiated populations from other continents, including Europe and Africa. Variants introduced into the indigenous population may have been positively selected for in subsequent epidemics. This is a distinct possibility given the widespread admixture found throughout the continent. A new method for detecting this type of selection utilizes haplotypes that are assigned to a particular ancestry. These haplotypes are then assessed in a probabilistic manner to examine whether their frequencies are more consistent with stochastic forces, such as demography, or deterministic forces, such as positive selection (Figure 3A). The use of such methods has yielded potential targets of positive selection that involve both the innate and adaptive immune response [33,34]. However, these findings fail to correlate the specific genes with pathogens that were introduced after European contact, which weakens arguments for selection by lacking a connection to a known environmental factor.

Lastly, considerable questions remain around the existence of human pathogens in the environments of South America before the arrival of Europeans. A common misconception depicts the Americas as a place without significant pathogens, as European colonizers brought disease with them but ostensibly did not suffer the reverse exposure to native pathogens from the Americas [35]. This observation has more to do with the speed and sheer abundance of pathogens presented to indigenous people by Europeans, coupled with the myriad social disruptions, including warfare, displacement, and slavery [35]. However, instances have been recorded where Europeans suffered devastating losses due to disease. One such record occurred during the French attempt at building the Panama Canal, where disease killed many of the French laborers. However, local indigenous people seemed immune to the various fevers afflicting the French, which were likely caused by arboviruses and flaviviruses being spread by mosquitos [36]. If this is true, then the indigenous people of Panama might display signs of strong positive selection on genetic variants that may correlate with the immune response to these types of viruses.

Bioarcheology also has the potential to reveal the presence of ancient human pathogens in South America. Ancient skeletons that exhibit pathologies may contain the genomes of the associated pathogens. The use of metagenomics, which extracts and aligns DNA sequences from ancient bone to the genomes of known microbes, may hold the key to revealing ancient disease. For the most part, research in this area has focused on detecting the presence of tuberculosis before European contact in the Andes [37], but the potential is high for revealing other endemic pathogens throughout South America.

## 6. Methods for Detecting Positive Selection

Testing the various hypotheses and scenarios posed in the article requires careful consideration of the populations utilized. Aside from requiring ancient samples with high endogenous DNA to reliably call genotypes, populations being compared across transects of time need to demonstrate genetic continuity. In other words, the researcher needs to be testing the same population through time when dealing with certain questions of adaptation. This is pertinent to questions concerning adaptation to pathogens introduced after European contact, where the researcher may test for positive selection before and after European contact (Figure 3B). On the other hand, it is possible to contrast genomic differences in ancient populations with known cultural or environmental differences to assess selection that is specific to a distinct environmental variable, which is seen in one population but not the other.

**Figure 4:**
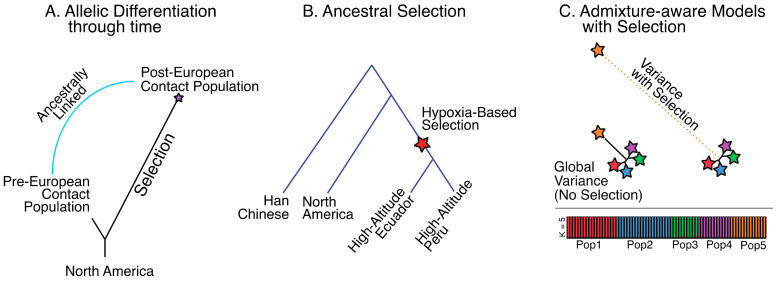
Various schemes for detecting selection in South American living and ancient populations. (**A**) Detecting allelic differentiation through time in a single population. (**B**) Detecting ancestral selection leading to adaptive phenotypes in two populations; (**C**) Detecting selection with admixture aware methods.

The population branch statistic (PBS) [11], a variant of the locus-specific branch length [38], lends itself well to this type of hypothesis testing, as it can handle juxtaposing a single population through time, as in the case of testing a population before and after European contact, or two distinct populations with different cultural or environmental attributes (Figure 4A). Depending on the hypothesis, PBS could detect strong selection that has occurred along one population but not the other since their divergence from a closely related outgroup. Careful attention also needs to be given to the outgroup, which could potentially obscure results, if the outgroup is too distantly removed from the comparative groups and has experienced a distinct evolutionary history.

Methods for detecting ancestral selection have also been developed. These methods employ both model-free approaches based on allele frequencies, which can detect shared ancestral selection between a set of populations since they diverged from a closely related outgroup (Figure 4B) (ancestral branch statistic (ABS) [39], the similar levels of exclusively shared differences (LSD) method [40,41], and the model-based 3P-CLR [42]). A wealth of additional approaches also exist for detecting shared ancestral selection events [43,44,45,46,47]. This collection of methods could potentially be utilized to test ancestral selection in ancient and living indigenous populations that share coastal environments and marine sustenance strategies. This test would be intriguing given that the first migrating people may have taken a coastal route into the Americas [48], potentially utilizing a marine diet along the way. Early South American archeological sites also show settlements along the South American Pacific coast [49], and these settlers are likely to be closely connected to the first migrants. One testable hypothesis is that the marine settlers came prepared with an adaptive phenotype that allowed them to thrive on the specific nutrients associated with a marine diet.

New methods are also available that directly compare populations without the need of an outgroup. One such approach is SS-H12 [47], which employs haplotype data. This method has great power to detect shared positive selection events, and excellent accuracy at distinguishing between events that occurred prior to population splits (ancestral selection) and independently in each population after their split (convergent selection). These two scenarios are difficult to disentangle with allele frequencies alone, and they lend insight into different interpretations of the adaptive history of the populations. This method could be useful in detecting independent adaptive events to similar agriculture products in different parts of South America. Another method employed by Ohana [15] is particularly dynamic because it involves the simultaneous assessment of admixture along with allelic differentiation (Figure 4C). In doing so, admixture between populations, if any exists, can be integrated into the test to prevent potential false positives or negatives. In a case where low- and high-altitude Andean populations are contrasted, Ohana could detect variants that deviate strongly in the high-altitude population with respect to a genome-wide covariance structure created using both coastal and high-altitude populations, which includes any admixed features. This method proved powerful in detecting selection on variants associated with the adaptive ability of deep sea divers in the indigenous Bajau people, who can hold their breath while diving for extraordinary amounts of time [50], and may provide the key for unlocking the hidden genetic components behind hypoxia adaptation in the Andes.

The approaches we have discussed thus far seek to measure and summarize genomic diversity with a single statistic, which is often engineered to detect a common footprint of some adaptive processes. However, more power and accuracy in detecting adaptive genomic targets may be acquired when examining multiple statistics in aggregate. In the last decade, advancements in computing power and artificial intelligence methodologies have fueled a renaissance in the development of new machine learning frameworks [51,52,53,54] that detect and quantify diverse evolutionary processes. These powerful approaches have been developed to identify diverse adaptive events [51,54,55], which include different mechanisms of positive selection and the estimation of key underlying evolutionary genetic parameters, such as recombination rates, population sizes changes over time, strengths of selection, frequencies of beneficial variants when selection is initiated, and ages at which variants became adaptive [52,56,57,58]. These methods can be utilized with large genomic datasets and multiple populations, allowing for hypothesis testing between populations with different attributes. Moreover, the varied modeling techniques employed by these methods permits them to directly account for the expected correlation structure among summary statistics across genomes (such as with Trendsetter [59] and SURFDAWave [60]), as well as to even automatically estimate genomic features from raw genotype calls that yield the highest prediction accuracies (such as with the methods of Flagel et al. [56], ImaGene [61], and BaSe [62]). One particular caveat of these methods is that an accurate demographic model is generally needed for the production of training data. Utilizing an ill-fitting model to the populations in question could potentially negatively influence the selection results. Luckily, recent work has provided significant insights on the demographic history of the South American continent [2,3,24], and studies have shown that training such machine learning models on diverse demographic histories may help rescue the negative effects of uncertain demographic history [59,60]. An additional caveat, however, remains in the “black box” nature of machine learning methods, which make it difficult to understand other evolutionary scenarios that could confound results.

While the above methods are powerful at detecting positive selection, the tests apply to instances where variants carry a very strong association to a particular phenotype. However, complex traits, which are governed by many variants and are complicated by environmental interactions, might also play a part in adaptive phenotypes. Moreover, the difficulties in identifying adaptation in genomic regions that are weakly associated with a particular phenotype, such as those related to complex traits, may be exacerbated when admixed populations are concerned, as is the case for methods that detect more classical signatures of positive selection. Though a number of frameworks have been developed to address such hurdles [63,64,65,66], we highlight one method known as PolyGraph [64], which implements a model-based approach to detect polygenic adaptation on complex traits that accounts for relationships among multiple putatively admixed populations using an admixture graph, and which could effectively be able to integrate genetic data from contemporary and ancient populations. This admixture graph is estimated from the genome-wide genetic variation within and across populations, which is taken as a null hypothesis of neutrality and attempts to account for the myriad demographic forces affecting such variation. PolyGraph has great power to pinpoint particular branches (either contemporary or ancestral populations) on the graph where adaptation may have occurred, and also has the ability to estimate strength of selection at each identified branch. Because demographic factors such as historical divergence and complex admixture history are directly incorporated, the method is able to uncover the subtle genomic footprints expected due to selection on complex traits.

## 7. Conclusions

The use of ancient DNA, and genomics in general, remains in its infancy with regard to understanding the evolutionary histories of the many populations of South America. Most of the research, to date, has been conducted in the Andes, in order to better understand the high-altitude phenotype and the complexities of the massive empires that existed before European contact. However, even in this region, many questions remain that can be addressed by future studies, including nuanced cases of adaptation and even epigenetic changes associated with cultural shifts. However, the Andes represents but one region of South America, which is filled with a great breadth of environments. These environments were successfully inhabited many thousands of years ago and little work has been done on the genomic level to understand the adaptive aspects of this success. To further complicate the issue, European contact dramatically shifted the population history of many cultures over the past 500 years, and the genomic impact of this shift is yet to be fully understood.
